# Gas-Sensing Devices Based on Zn-Doped NiO Two-Dimensional Grainy Films with Fast Response and Recovery for Ammonia Molecule Detection

**DOI:** 10.1186/s11671-015-1170-2

**Published:** 2015-12-01

**Authors:** Jian Wang, Xiaowei Wei, Peihua Wangyang

**Affiliations:** School of Materials Science and Engineering, Xihua University, Chengdu, 610039 People’s Republic of China; Information Materials and Device Application Key Laboratory of Sichuan Provincial Universities, College of Optoelectronic Technology, Chengdu University of Information Technology, Chengdu, 610225 People’s Republic of China

**Keywords:** Zn-doped NiO, Ammonia-sensing materials, Gas-sensing device, Response and recovery

## Abstract

Zn-doped NiO two-dimensional grainy films on glass substrates are shown to be an ammonia-sensing material with excellent comprehensive performance, which could real-time detect and monitor ammonia (NH_3_) in the surrounding environment. The morphology and structure analysis indicated that the as-fabricated semiconductor films were composed of particles with diameters ranging from 80 to 160 nm, and each particle was composed of small crystalline grain with a narrow size about 20 nm, which was the face-centered cubic single crystal structure. X-ray diffraction peaks shifted toward lower angle, and the size of the lattice increased compared with undoped NiO, which demonstrated that zinc ions have been successfully doped into the NiO host structure. Simultaneously, we systematically investigated the gas-sensing properties of the Zn-doped NiO sensors for NH_3_ detection at room temperature. The sensor based on doped NiO sensing films gave four to nine times faster response and four to six times faster recovery speeds than those of sensor with undoped NiO films, which is important for the NiO sensor practical applications. Moreover, we found that the doped NiO sensors owned outstanding selectivity toward ammonia.

## Background

In recent years, air pollution have become one of the most serious problems that every country in the world is facing, which also have attracted considerable attention from a part of the scientific community to investigation of various atmospheric environmental issues [1–5]. Ammonia (NH_3_) is one of the toxic gases in the earth’s atmosphere and is considered as an artificial source for intensive livestock breeding with the decomposition process of manure and the chemical industry for the production of fertilizers and for refrigeration systems [6, 7]. Generally speaking, upon exposure to around 50 ppm (corresponding to about 40 μg/m^3^), NH_3_ is irritating to respiratory system, skin, and eyes and could cause acute poisoning or life-threatening situations [8–11]. Therefore, it became highly desirable to design and fabricate a portable sensing device with low cost and low power, which can real-time detect and monitor NH_3_ in air [12]. Currently, some miniaturized chemical gas sensors based on nanoscale metal oxide semiconductor, which rely principally on monitoring the direct change in the conductance in adsorption and desorption of gas molecules, have been successfully obtained [13–15]. Gas-sensing performance strongly depends on the dimension, size, and morphology of the sensing materials. Thus, further research on sensing materials is still necessary for enhancing the sensing properties and continuing the expansion of the area of application of the sensor.

During the past several years, various semiconductor metal oxides such as WO_3_, CuO, TiO_2_, SnO_2_, ZnO, and In_2_O_3_ have been successfully prepared and their gas-sensing properties have been investigated from a part of the gas-sensing community [16–21]. Among these gas-sensing materials, naturally p-type NiO semiconductors with a wide band gap energy in the range of 3.6–4.0 eV have been recognized as an interesting and prosperous nanomaterials for the detection of gas molecules due to their simplicity of use, high compatibility with microelectronic processing, and environmental friendliness [22]. Recently, one of the most significant breakthroughs in the domain of gas-sensing devices based on metal oxides was the preparation of the doped-nanomaterials [23–25]. Specifically, various catalysts (such as Pt, Ag, and NiO) have been loaded into metal oxide-sensing materials to improve gas-sensing performances. The doping contents, distribution, and size of catalysts are key parameters for enhancing gas molecules response/recovery speeds as well as selectivity [26]. Therefore, doping metal ions in nanoscale NiO two-dimensional grainy films is considered as an effective and simple way to improve the gas-sensing properties (such as decreasing the response and recovering time) via forming micro area p-n junction (e.g., p-type NiO/n-type ZnO), which is very important for the application of gas sensors.

In our previous study, we developed a gas sensor based on pure NiO films for sensing NH_3_, which owned excellent stability and outstanding sensitivity performance [27]. However, as being a product, the major issue is that the recovery speed is very poor at room temperature because the desorptions of gas molecules adsorbed on sensor need take a long time, and similar problem was proposed via other investigators [28–30]. To solve this problem, we would fabricate the nickel oxide two-dimensional grainy films and doping zinc ions forming in micro region, which enhanced the response and recovery speeds via improving electron transport and gas molecular diffusion in this special nanostructure. In the present work, we present a hydrothermal controllable route combined with high-temperature oxidation approach to prepare Zn-doped NiO films with well-controlled morphologies and systematically investigate Zn-doping effects on the gas-sensing performance of sensors at room temperature. The results showed that the response and recovery speed of NH_3_ can be remarkably improved via doping zinc ions in nanoscale NiO films as compared with pristine NiO films, which may provide a simple approach to fabricate excellent gas sensors based on metal oxides with desirable gas-sensing performance.

## Methods

### Preparation of the Gas-Sensing Materials

**Fig. 1:**
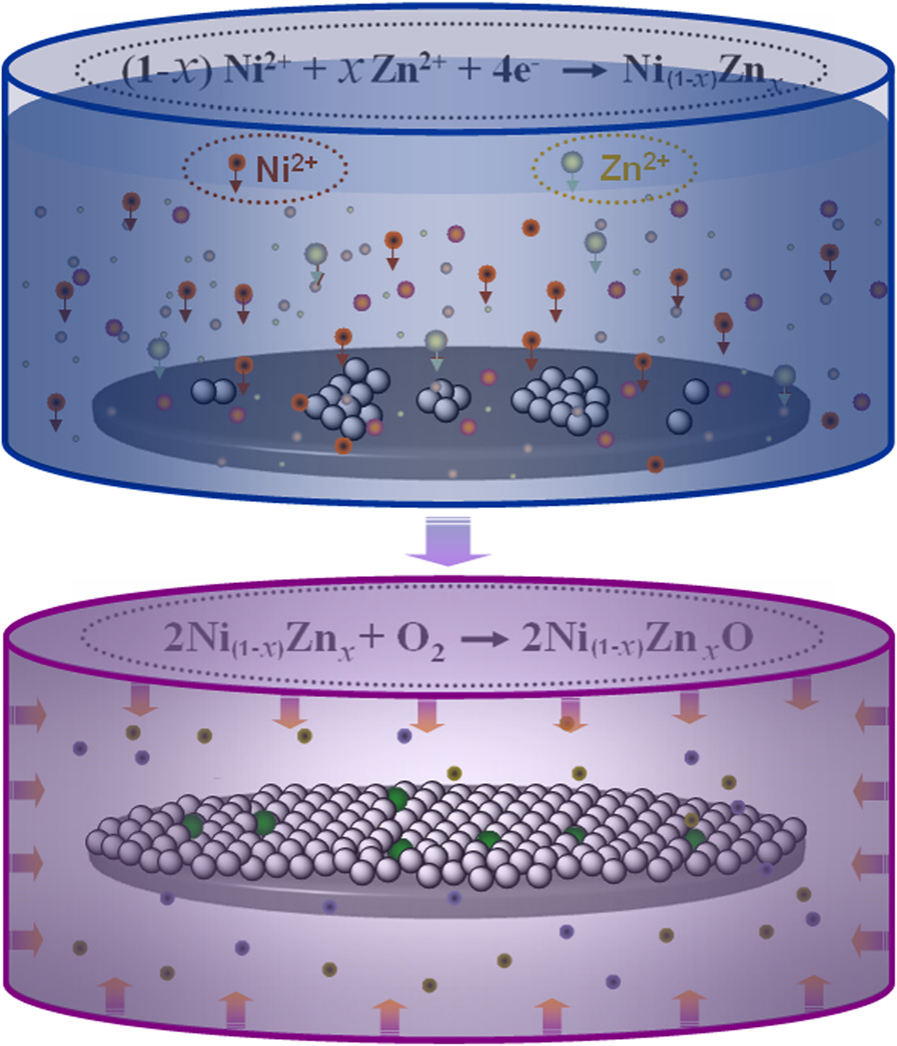
Fabricating schematic diagrams of the gas-sensing materials. Schematic diagrams show the specific method of synthesis of Zn-doped NiO films on glass substrates

The specific strategies to synthesize Zn-doped NiO films on glass substrates, where the thickness of substrate was about 2 mm, were summarized as follows. In a typical experiment, 7.14 g of nickel chloride hexahydrate (NiCl_2_·6H_2_O purchased from Sinopharm Chemical Reagent Company Limited) and a certain amount of zinc chloride (ZnCl_2_ purchased from Sinopharm Chemical Reagent Company Limited) with different molar ratios (ZnCl_2_ to NiCl_2_·6H_2_O), such as 0:100, 1:100, 3:100, 6:100, were dissolved into 200-mL distilled water under continuous magnetic stirring at room temperature for 15 min. Ultrasound treatment was carried out for 30 min to ensure that nickel and zinc ions were dispersed homogeneously in the water solution and yield a homogeneous grass green transparent solution. Then, 50-mL water solution of sodium hydroxide (NaOH purchased from Shanghai Chemical Reagents Company) and 25-mL water solution of hydrazine hydrate (N_2_H_4_·6H_2_O purchased from Sinopharm Chemical Reagent Company Limited) were dropwise into the above solution. This as-configured mixture was stirred constantly for 30 min, and the color of solution changed from grass green to homogeneous navy blue color. At this point, as-prepared mixture was transferred into the flask containing a glass substrate which was ultrasonically washed several times with ethanol and distilled water and subsequently heated to a constant temperature for a definite time at atmospheric pressure. After the reaction was completed, the resulting dark gray product covering of glass substrate was washed several times using ethanol and distilled water, respectively, and then the samples were dried in a vacuum oven at 100 °C for 5 h. Following that, the as-fabricated films were heated to 500 °C and kept the temperature constant for 6 h in a furnace in open air. The gray green products on the substrate obtained from above experiment were collected for future analysis. Figure 1 shows the flowchart of fabrication of the Zn-doped NiO films on glass substrate.

### Assembling of the Gas-Sensing Devices

**Fig. 2:**
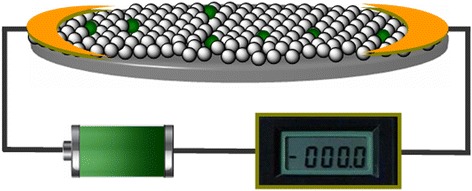
Configuration of gas-sensing devices. Schematic diagrams show the configuration of gas-sensing devices, which mainly contained two electrodes and the Zn-doped NiO film gas-sensing materials

For the fabrication of the gas-sensing devices, the as-prepared Zn-doped NiO films on glass substrate was used as the gas-sensing materials. Specific assembling procedure and the configuration of gas sensors were shown in Fig. 2. Step one: the edges of as-prepared Zn-doped NiO films were sputtered with 30 nm pure titanium and, subsequently, were connected with the positive and negative electrodes using the homemade silver paste and dried in the vacuum oven at 90 °C for 2 h. Step two: the edge-sputtered titanium of gas-sensing materials were continued to be sputtered with 130-nm pure gold using a patterned mold in a magnetron sputtering apparatus. Step three: the as-fabricated electrodes were bound to the gas-sensing sockets using micrometer scale gold wires in nanoscale welding machine.

### Characterization

The crystallinity and crystalline nature of each synthesized crystal were identified via X-ray powder diffraction (XRD) using a 18-kW advanced X-ray diffractometer (D8 ADVANCE, Bruker, Germany) in a two theta range from 30 to 90° with a Cu Kα (*λ* = 0.154056 nm) rotating anode point source operating at 40 kV and 40 mA. The specific surface morphology and elemental composition of as-prepared samples were investigated using a field emission scanning electron microscopy (FESEM, Zeiss Ultra 55, Germany) combined with energy-dispersive spectroscopy (EDS) at accelerating voltage of 5 and 20 kV, respectively. In addition, high-resolution transmission electron microscopy (HRTEM) images were recorded in a JEM-2010 transmission electron microscope operating at 200 kV. The electrical signal of the gas-sensing devices was monitored using the Agilent 4156C. The gas-sensing properties of sensor were evaluated via the resistance change at a working voltage of 1 V.

## Results and Discussion

### Structure and Morphology of Gas-Sensing Materials

Zn-doped NiO films on glass substrate were fabricated via the hydrothermal reaction combined with subsequent high-temperature oxidation method, as shown in Fig. 1. In the first stage of synthesis of gas-sensing materials, a controlled hydrazine reduction approach has been developed to synthesize Ni_(1 − *x*)_Zn_x_ films on a substrate in a flask. In the second stage, Ni_(1 − *x*)_Zn_x_ films were transformed Ni_(1 − *x*)_Zn_x_O films via situ chemical oxidation route in open air. The specific chemical reactions for the synthesis can be expressed as follows:

Step 1:

1 $$ \left(1-x\right){\mathrm{Ni}}^{2+}+x{\mathrm{Zn}}^{2+}+4{\mathrm{e}}^{-}\to {\mathrm{Ni}}_{\left(1-x\right)}{\mathrm{Zn}}_x $$

Step 2:

2 $$ 2{\mathrm{Ni}}_{\left(1-x\right)}{\mathrm{Zn}}_x+{\mathrm{O}}_2\to 2{\mathrm{Ni}}_{\left(1-x\right)}{\mathrm{Zn}}_x\mathrm{O} $$

**Fig. 3:**
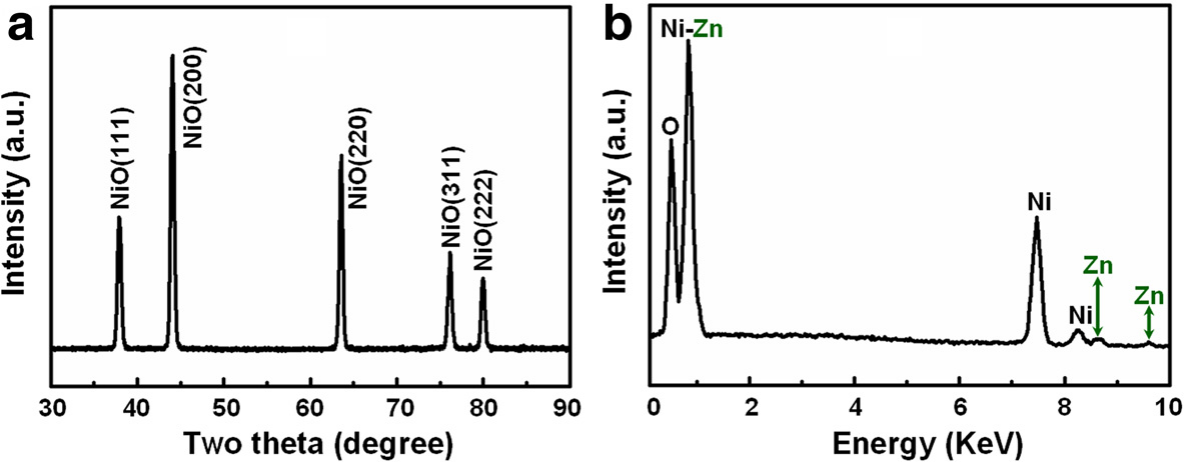
XRD and EDS analysis of Zn-doped NiO film. **a** XRD pattern of the Zn-doped NiO films. **b** EDS analysis confirming that the as-prepared products are composed of only Ni, Zn, and O element

In the characterization of gas-sensing materials, we found that the X-ray powder diffraction (XRD) patterns of the as-prepared gray green films with and without the introduction of zinc ions are similar. Figure 3a shows the XRD pattern of the as-fabricated 1 mol % Zn-doped NiO film. All diffraction peaks in this pattern were found to match with pure NiO phase with cubic structure, which was consistent with the value given in the standard card 47-1049. Specifically, the peaks at the scattering angles (i.e., two theta) correspond to the crystal planes (111), (200), (220), (311), and (222) of crystalline NiO. From the XRD pattern of Fig. 3a, no other impurity peaks (such as nickel, nickel hydroxide, zinc, zinc oxide, and zinc hydroxide) were detected, which indicated that the single cubic structure was obtained in as-prepared gas-sensing film. In order to confirm zinc doping, further EDS analysis of film revealed the peaks of nickel, oxygen, and zinc, which illustrated that as-prepared film was composed of only Ni, O, and Zn. The similar atomic ratios of Ni, O, and Zn were observed at five different points of film, and the average value of Zn content was about 0.93 %, which is basically consistent with the Zn-doping concentration (1 mol%) in our experiment.

**Fig. 4:**
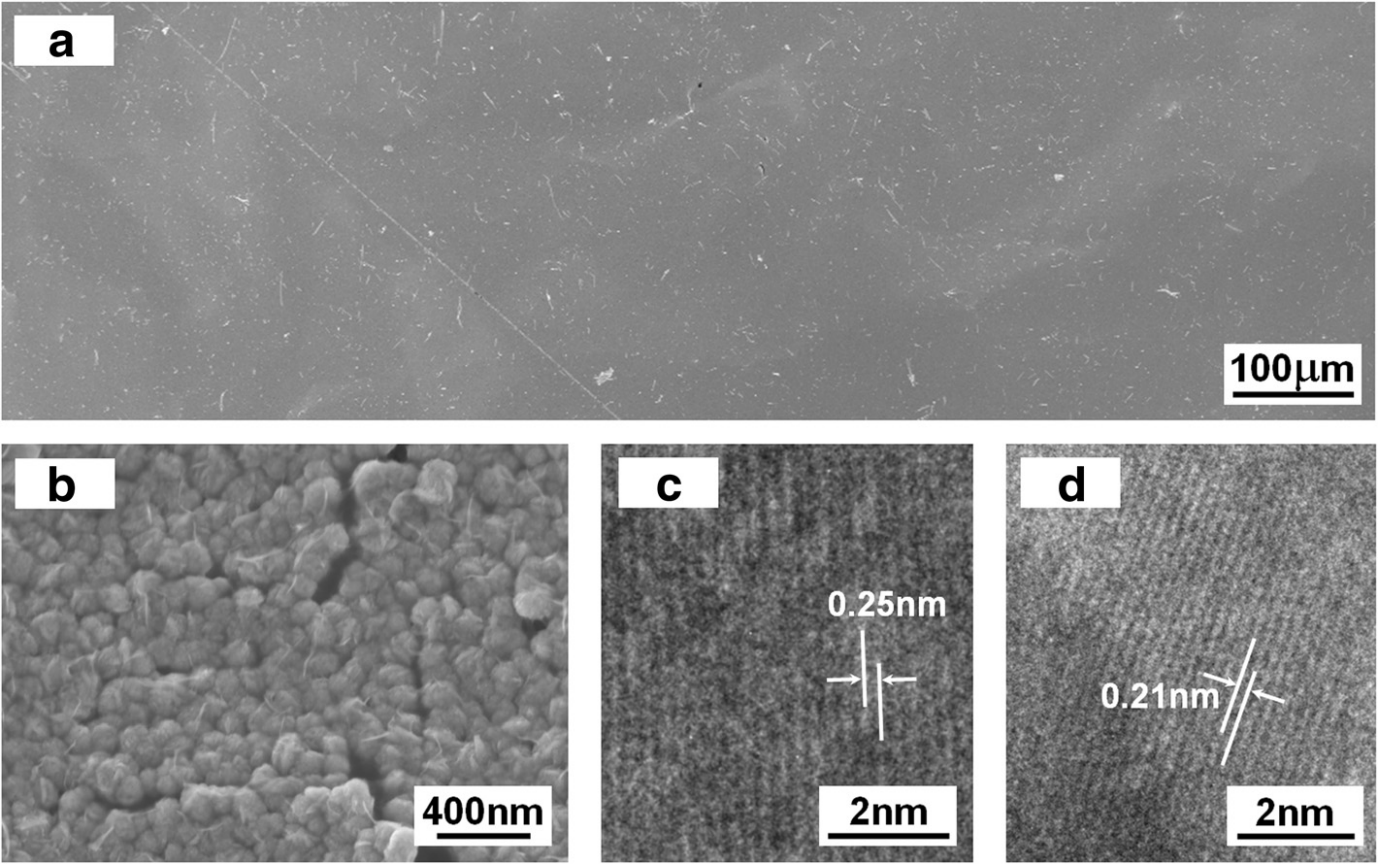
TEM and HRTEM analysis of Zn-doped NiO film. **a** Low-magnification and **b** high-magnification SEM images of sample demonstrating Zn-doped NiO film is composed of NiO particles. **c**, **d** HRTEM images recorded from two different areas of sample

The crystallinity, crystal size, and porosity are three most important factors that affect the gas sensitivity of metal oxide thin film devices. In XRD patterns, the sharp and strong peaks indicate that the products are well crystallized. According to the Scherrer formula from the full width at half maximum (FWHM) of (111), (200), (220), (311), and (222), the average crystalline grain size of the sample is about 20 nm, and the standard deviation (8 nm) of grains have been calculated via measuring more than 50 points from different samples. Figure 4a presents the scanning electron microscopy (SEM) image of the as-prepared 1 mol % Zn-doped NiO film distributing all over the glass substrate. The morphology of sample is a two-dimensional grainy film with an average of 300 nm thickness. The high-magnification image in Fig. 4b clearly demonstrates the doped NiO film has a uniform distribution of particles with diameters ranging from 80 to 160 nm, which is obviously larger than the crystalline grain size calculated from X-ray diffraction peaks, illustrating that the particles were composed of many crystalline grains. And this film is highly ordered porous morphology, which is very important for the physical adsorption of gas molecules.

In order to understand the internal microstructure of the doped film in more detail, further HRTEM images recorded from a particle in film are shown in Fig. 4c, d, which shows the crystal lattice structure. The images with no visible or planar defects show the high crystallinity of the Zn-doped NiO film. The inter-planar distance of the fringes in Fig. 4c was measured to be 0.245 (approx. 2.5) nm, which is slightly larger than that for the (111) plane of intrinsic cubilc NiO crystal (0.24120 nm). In the meantime, the interspacing in Fig. 4d was determined to be 0.212 (approx. 2.1) nm, which is also slightly larger than that for the distance of intrinsic NiO (200) plane (0.20890 nm). These results indicate lattice expansion, which implied that zinc atoms might form substitution atoms in NiO film to a great extent.

**Fig. 5:**
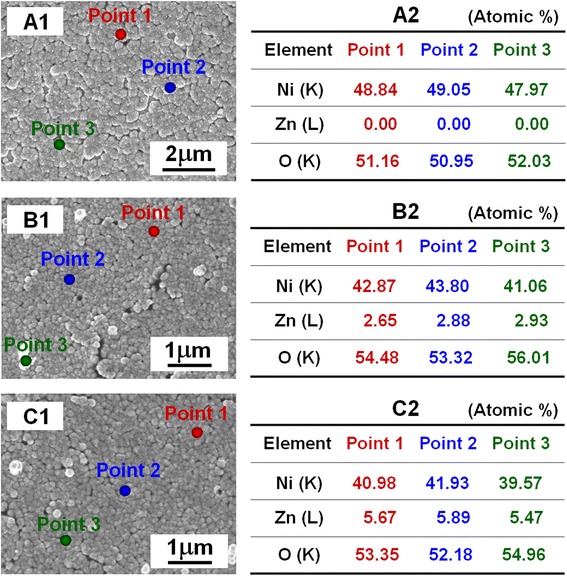
SEM and EDS analysis of NiO films doped with different concentrations of Zn. **A1–C1** SEM images of NiO films with 0, 3, and 6 mol % of Zn-doping concentration. **A2–C2** EDS analysis from a series of locations on films was labeled with point 1, point 2, and point 3, and the quantitative element distribution in atomic percentage is tabulated

The evolution of the surface morphology and element distribution of the Zn-doped NiO films in different Zn-doping concentrations have been investigated and are shown in Fig. 5. Figure 5A1–C1 show SEM images of as-prepared NiO films with 0, 3, and 6 mol % of zinc-doping concentration. The two-dimensional grainy structures remained similar after the NiO films were doped with different concentrations of Zn, which indicated that the Zn dopant (at 0–6 mol % doping concentration range) has no influence on the growth of the gas-sensing films. The point-EDS spectral microanalysis from a series of locations on the films was labeled with points 1, 2, and 3 (the solid red, blue, and green dots, respectively) in Fig. 5A1–C1, and the quantitative element distribution in atomic percentage is tabulated in Fig. 5A2–C2. 0.00, 2.65, and 5.67 at. % Zn were measured, as shown in point 1 of Fig. 5A2–C2, which is basically consistent with the Zn ions doping concentration (0, 3, and 6 mol %), respectively. Similar Zn contents were observed at various points of films, such as point 2 and point 3.

**Fig. 6:**
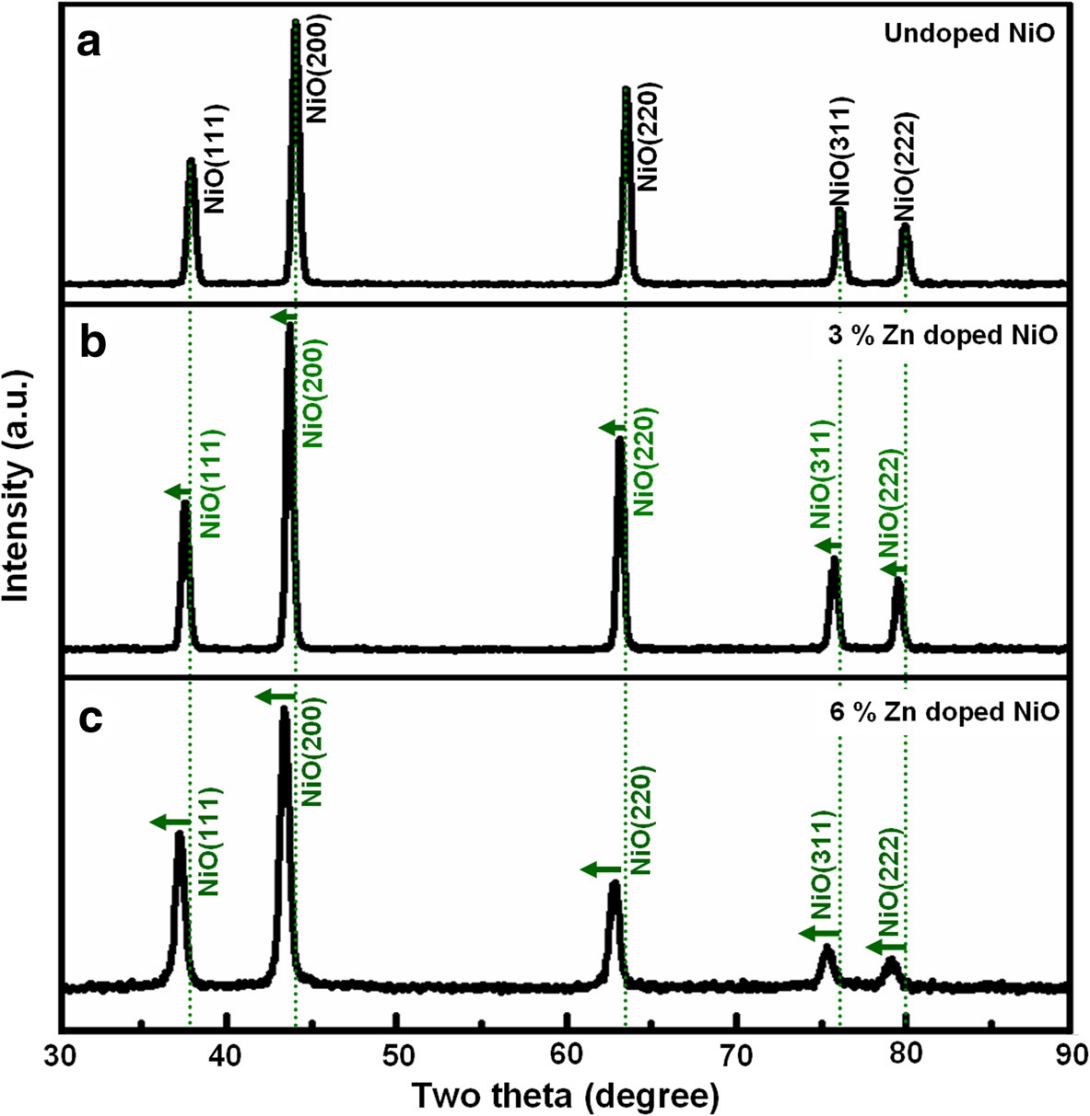
XRD analysis of NiO films doped with different concentrations of Zn. XRD patterns of **a** undoped, **b** 3 %, and **c** 6 % Zn-doped NiO films

The corresponding XRD patterns of the three samples with various levels of Zn doping are presented in Fig. 6. In “0 mol% Zn-NiO,” “3 mol % Zn-NiO,” and “6 mol % Zn-NiO,” the observed X-ray diffraction peaks can be indexed to a standard cubic NiO structure with no visible defects, secondary phases, or impurity peaks. These results suggest that the dopants are well-integrated into the NiO lattice sites during the growth process. However, all the peaks of 3 mol % Zn-NiO and 6 mol % Zn-NiO have a slight shift toward lower angles with increasing Zn dopant concentration compared with 0 mol % Zn-NiO samples, which is assigned to the successful incorporation of zinc ions in the NiO host structure. The larger ionic radius of the zinc ion (0.074 nm), compared with the nickel ion (0.069 nm), tends to increase the size of the lattice in doped NiO films, which is consistent with the HRTEM observation in Fig. 4c, d.

### Gas-Sensing Performances of the Sensors

As well as we know, the stoichiometric NiO crystal is an insulator, however, typical NiO exhibits a reasonable electrical conductivity due to nickel vacancies or interstitial oxygen atoms in NiO crystal (p-type semiconductor) [31]. As-synthesized Zn-doped NiO film exhibited oxygen-rich stoichiometry (see Fig. 5A2–C2), such as the atomic ratio O/Ni of 0 mol% Zn-NiO was about 51.16/48.84. Ammonia-sensing devices based on doped NiO films were fabricated via connecting the gas-sensing materials to the testing sockets using homemade silver paste combined with subsequent sputtering pure Au, as shown in Fig. 2. In our testing experiments, the conductance between the positive and negative electrodes was measured with a precision semiconductor parameter analyzer (Agilent 4156C) to investigate the response of gas-sensing devices. The sensor response is defined via the equation (ΔG/G_0_ = (G–G_0_)/G_0_), where G_0_ and G are the conductance of sensing films when exposed to the air and target gas with 25 % of relative humidity, respectively. The response time (τ_response_) and recovery time (τ_recovery_) are defined as the time needed to reach 90 % of the final steady state conductance upon exposure to the testing gas and air, respectively [32].

**Fig. 7:**
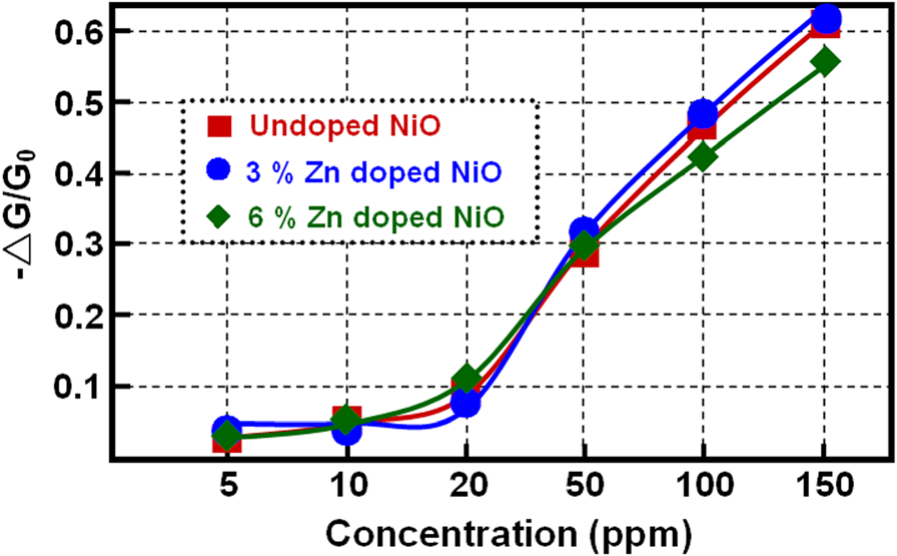
Sensitivity tests of the gas sensors based on three types of NiO films. The conductance changes of gas-sensing materials toward different concentrations of NH_3_ at room temperature

Figure 7 shows the gas response of the Zn-doped NiO including a pure NiO film and the two Zn-doped NiO films with 3 % and 6 mol % Zn based gas-sensing devices, when exposed to different concentrations of NH_3_ at room temperature with 25 % of relative humidity. There were no significant changes in the sensor response after doping the NiO films with 3 % and 6 mol % Zn. Meanwhile, the response change in a series of sensing cycle experiments with different concentrations of NH_3_ from 5 to 150 ppm was investigated, as seen in Fig. 7. Obviously, the change of response increased with the increasing NH_3_ concentrations, but the change in response after doping the NiO films remained similar for a certain amount of NH_3_, which indicated that the Zn dopant has no remarkable influence on the sensitivity (response intensity) of the sensing device. This phenomenon could be because the three samples have a similar grain size and number of grain boundaries after the same high-temperature oxidation conditions. Previous reports have demonstrated that the grain boundaries or grain junctions (large porosity and crystal defects) were considered to be active sites for gas molecule absorption and affect electron transport through the sensing films, resulting in conductance change [26].

**Fig. 8:**
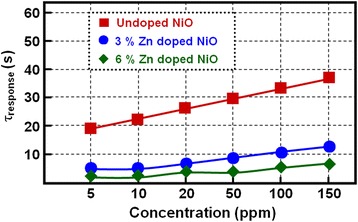
Response tests of the gas-sensing devices. Response time tests of sensors based on three types of NiO films to different concentrations of NH_3_ at room temperature

**Fig. 9:**
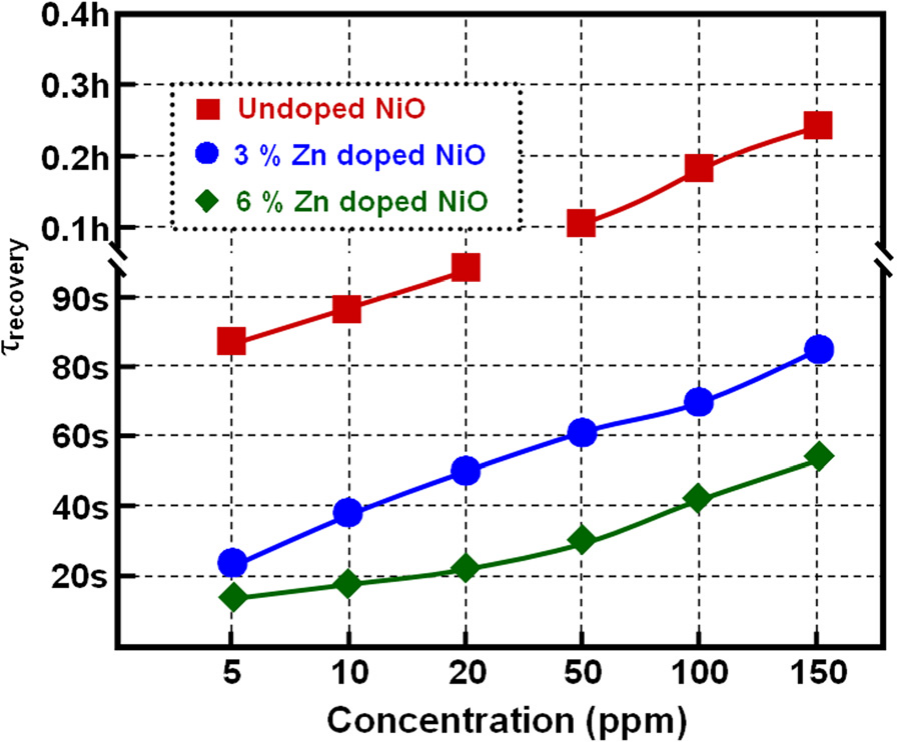
Recovery tests of the gas-sensing devices. Recovery time tests of sensors based on three types of NiO films to different concentrations of NH_3_ at room temperature

In the undoped NiO film based gas sensor, the response time (about 19 s, Fig. 8) and recovery time (about 83 s, Fig. 9) are very long upon exposure to 5 ppm NH_3_, which is not favorable for the NiO film-based sensor practical applications. This slow response and recovery speed could be attributed the slow surface reactions consisting of the adsorption, dissociation, ionization, and diffusion of gas molecules [14]. In contrast, the response time and recovery time of the gas sensor became very short when doping with 3 and 6 mol % of Zn, which means that the response speed was increased by about four to nine times and the recovery speed was increased by about four to six times via doping Zn in NiO sensing films. This fast response speed demonstrates that both the in-diffusion of NH_3_ to the surface of Zn-doped NiO crystals and its electrochemical oxidation by reaction with negatively charged oxygen species occur very quickly. Additionally, this fast recovery speed suggests that the doping Zn plays an important role in shortening the recovery reaction involving the dissociation, ionization, diffusion, and desorption of the oxygen species and ammonia molecules in reference gas (air) blowing [33]. In the meantime, the response and recovery speed also showed slightly different behavior for NiO films doped with different concentrations of Zn. For example, the response and recovery time of the 3 mol % Zn-NiO sensor was 5 and 22 s, and the 6 mol % Zn-NiO sensor was 2 and 14 s upon exposure to 5 ppm NH_3_, respectively, which shows that an increase in the concentration of Zn speeds up the response and recovery speed. However, when the Zn concentration surpassed 6 mol % (i.e., solubility limit) in NiO sensing films, the response and recovery speeds remain constant. This phenomenon could be explained through the following sensing mechanism. Zn-doped NiO films own more electrons owing to n-type ZnO crystallites and could quickly adsorb oxygen and then speed up the response speed of gas sensor. Ammonia could easily react with oxygen, and the electrons quickly returned back into the sensing films, that is to say, speeding up the recovery speed.

**Fig. 10:**
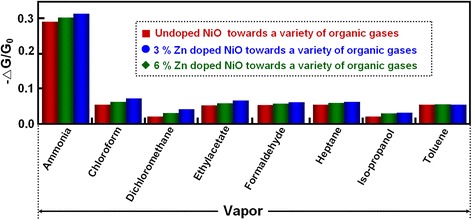
Selectivity tests of the gas sensors based on Zn-doped NiO films. The concentration of ammonia is 50 ppm, and the concentration of other organic gases is higher than 300 ppm

Selectivity is a very important parameter for utility-type metal oxide gas-sensing devices, which relies mainly on the specific interactions between the sensing materials and the gas molecules [26]. The response of gas sensor based on Zn-doped NiO films toward a variety of toxic, corrosive and flammable gases including ammonia, chloroform, dichloromethane, ethylacetate, formaldehyde, heptane, iso-propanol, and toluene were explored to evaluate the selectivity, as shown in Fig. 10. Remarkably, this gas sensor exhibited excellent selectivity to NH_3_ when exposed to these interfering gases. For example, the sensitivity of the sensors based on NiO films in different Zn-doping concentrations to 50 ppm of NH_3_ was about 30 %, whereas that sensitivity was less than 8 % to higher concentration (>300 ppm) of other organic gases. Furthermore, compared with undoped NiO film based sensor, the response of 3 mol % Zn-NiO sensor presented an increase and reached its maximum value of 31 % toward 50 ppm of NH_3_ and then decreases with further increase of Zn-doping concentration. Meanwhile, the gas sensors based on Zn-doped NiO films exhibited excellent stability and repeatability in our test experiments. These results suggest that the assembling NiO film is an excellent strategy for promoting the selectivity of the gas-sensing devices and controlling Zn-doping concentration could further improve selectivity.

## Conclusions

In summary, semiconductor Zn-doped NiO gas-sensing films on glass substrates have been successfully fabricated via a chemical reaction combined with subsequent high-temperature oxidation method. SEM observations revealed that the doped NiO film composed of nanoscale particles with diameters ranging from 80 to 160 nm, which was larger than the crystalline grain size (about 20 nm) calculated from X-ray diffraction peaks; and Zn-doping concentrations had no influence on the growth of the gas-sensing films. In XRD and HRTEM characterization of doped NiO films, we found that all the XRD peaks shifted toward lower angle and the size of the lattice increased compared with undoped NiO samples, which is assigned to the successful incorporation of zinc ions in the NiO host structure. Significantly, the gas-sensing devices based on as-fabricated Zn-doped NiO films exhibited the short response/recovery time, excellent sensitivity, and selectivity toward ammonia over other organic gases, such as chloroform, dichloromethane, ethylacetate, formaldehyde, heptane, iso-propanol, and toluene. It is suggested that the approach demonstrated here could also be extended to other element-doped sensing films for corresponding gas-sensing devices, which realize gas detection at room temperature.
